# Production and use of regional climate model projections – A Swedish perspective on building climate services

**DOI:** 10.1016/j.cliser.2016.06.004

**Published:** 2016-09

**Authors:** Erik Kjellström, Lars Bärring, Grigory Nikulin, Carin Nilsson, Gunn Persson, Gustav Strandberg

**Affiliations:** a Swedish Meteorological and Hydrological Institute, S-60176 Norrköping, Sweden; b Centre for Environmental and Climate Research, Lund University, S-223 62 Lund, Sweden

**Keywords:** Climate services, Regional climate modelling, Sweden, CORDEX

## Abstract

We describe the process of building a climate service centred on regional climate model results from the Rossby Centre regional climate model RCA4. The climate service has as its central facility a web service provided by the Swedish Meteorological and Hydrological Institute where users can get an idea of various aspects of climate change from a suite of maps, diagrams, explaining texts and user guides. Here we present the contents of the web service and how this has been designed and developed in collaboration with users of the service in a dialogue reaching over more than a decade. We also present the ensemble of climate projections with RCA4 that provides the fundamental climate information presented at the web service. In this context, RCA4 has been used to downscale nine different coupled atmosphere-ocean general circulation models (AOGCMs) from the 5th Coupled Model Intercomparison Project (CMIP5) to 0.44° (c. 50 km) horizontal resolution over Europe. Further, we investigate how this ensemble relates to the CMIP5 ensemble.

We find that the iterative approach involving the users of the climate service has been successful as the service is widely used and is an important source of information for work on climate adaptation in Sweden. The RCA4 ensemble samples a large degree of the spread in the CMIP5 ensemble implying that it can be used to illustrate uncertainties and robustness in future climate change in Sweden. The results also show that RCA4 changes results compared to the underlying AOGCMs, sometimes in a systematic way.

**Practical implications**

Climate information derived from an ensemble of simulations with the Rossby Centre regional climate model (RCA4) is the foundation of the climate service presented here. A central facility is the material presented at the SMHI climate scenario web pages (http://www.smhi.se/en/climate/climate-scenarios) that has been in operation since the start of October 2013. The actual content and format of what is displayed at the web site has been developed during the last decade in an iterative process involving a close dialogue with a range of users of the services as described in more detail in this study. Here, we first give a short description of what is currently published on the web page that presents both traditional climate change information in the form of maps and diagrams but also more detailed information on what is shown and guidance documents on how the results could be interpreted and further used. There are also links that can be used to download the data. The displayed material is stratified along several dimensions: area, forcing scenario, seasons and climate index. For each of these there are a number of options for what can be displayed at the screen by a user.

In the dimension of area, results from the global scale down to the local scale are presented. At the global scale information from the underlying ensemble of global climate models, which have been used as input data to the more detailed regional model, is used to produce the maps presented. By looking at these maps one can get consistent information about how the regional and local climate change signal compares to that in other areas of the world. At the global level focus lies only on seasonal mean temperature and precipitation. For the European and Swedish areas results from RCA4 have been used. At the Swedish level, which contains most information, data can also be displayed in diagram form as averages for different regions (all country, administrative counties, weather forecast districts, main catchment areas). For Swedish conditions also observational data are shown. This allows the user of the web site to get an idea about the observed interannual variability of the displayed climate index in the region. This can then be considered in relation to the future variability as projected by the climate model.

Forcing scenarios include both the newer generation of RCPs (representative concentration pathways) being used in the most recent IPCC assessment reports (IPCC, 2013) and older Special Report of Emission Scenarios (SRES, Nakićenović et al., 2000) used in earlier IPCC assessment reports. By displaying results from different generations of scenarios users of climate information can compare between what they have used previously with the more recent information.

Currently, 14 different indices, as listed in Table 1, are shown in the maps for the four seasons and for annual mean conditions. The indices have been chosen as they; i) are of interest to the users as they typically have some impact and ii) that they represent features for which RCA4 performance has been evaluated against the observed climate. We note here that this does not imply that the model results are perfectly matching the observations but that we have a good picture of how large the biases may be. It is clear from the results that the inclusion of indices that take into account length of certain periods or relation to specific thresholds broadens the picture of the changing climate compared to simpler indices only taking into account direct changes in the underlying variables (e.g. average change in temperature, change in maximum daily precipitation amount).

Data are presented both as ensemble means and in terms of spread between the different RCA4 runs (Fig. 1). The spread is given as the standard deviation calculated from the nine different runs. Also maps indicating how many out of the nine ensemble members that show positive changes in an index are displayed. Taken together this information can be used to assess the main direction and amplitude of climate change as well as the spread around the central value and also give an indication of the robustness of the results.

After a few years of operation it stands clear that the climate service described here and provided through the SMHI web site is of good practical use in the Swedish work on adaptation to climate change. This has been indicated by feedback from the Swedish County administrative boards that are responsible for regional coordination of climate change adaptation in Sweden. We can also note that the web service has a high rate of access (Fig. 2) with more than 125.000 exclusive page views since its launch in October 2013. The time line of Fig. 2 shows that the web service is accessed throughout the year, albeit with a minima in the summer (vacation) period. It also indicates that the usage is larger at some points in time coinciding with certain events or promotional activities. The most prominent ones include: the launch of the web service (October 2013), launch of new RCP2.6 scenarios (November 2014), presentation of governmental assignments and publishing of a user guide for climate scenarios (December 2014), launch of a +2 °C scenario (November 2015) and the COP21 climate meeting in Paris (December 2015). In addition, our experience is that the material is most useful in contacts with journalists.

## 1 Introduction

As a designated national expert agency for weather, climate, hydrology, and oceanography in Sweden, SMHI has a long experience of communicating with a wide range of users. With the raising awareness of climate change and its impacts a need has emerged for “actionable” information on climate and climate change (Asrar et al., 2013). To meet these new challenges SMHI activities pertaining to climate and climate change communication has over the last decades evolved to not only *inform* society about weather and climate, and *discuss* the information *after it has been presented*, but to more actively *involve the users* of that information already in *the early stages of production and design* of the presentation material.

In particular, three milestone events have shaped the development of SMHI’s climate change information activities over the last two decades: i) in 1997 the SWECLIM research programme was initiated and the Rossby Centre was formed at SMHI as central hub for building capacity for regional climate model research and development. As a result of this research programme, the Rossby Centre built a viable capacity in regional climate modelling (Rummukainen et al., 2004) thus providing the scientific foundation for the production of climate change information; ii) in 2005 the Government appointed the Swedish Commission on Climate and Vulnerability that initiated a broad cross-sectoral assessment of climate vulnerability (SOU, 2007). SMHI worked closely with the Commission and its working groups to provide extensive information and expert support and through this process built substantial experience in communicating climate change information with a broad spectrum of stakeholders; iii) in 2011 the Coordinated Regional Downscaling Experiment (CORDEX, initiated in 2009) began to gain momentum (Jones et al., 2011). To meet the increasing need for production and publication of regional climate scenarios outlined by CORDEX the Rossby Centre developed a more streamlined technical production process permitting multiple ensembles of scenarios to be produced and presented. After this brief historic overview we now introduce the different stages and their role in forming the current provision of climate services and its uptake within Sweden.

Over the years, a number of coupled model intercomparison projects (CMIPs) have produced a vast amount of global climate model (GCM) results that can be used to assess possible future climate changes (e.g. Meehl et al., 2007, Taylor et al., 2012). However, the GCMs operate on relatively coarse horizontal resolution implying that regional details of topographic features including land-sea distribution, vegetation cover and altitude of the terrain are described with relatively few details. Furthermore, some relevant atmospheric processes, including mid-latitude and tropical cyclones, are only crudely represented, and finer-scale phenomena are not resolved at all. Regional climate models (RCMs), operated on limited area domains at higher horizontal resolution can to a better extent simulate so called synoptic and meso-scale processes. Consequently, RCMs have been put forward as a means of producing information on scales closer to where actionable information is needed (e.g. Rummukainen, 2010).

Development and application of the Rossby Centre regional climate model (RCA) has been a focus area of the Rossby Centre since it was established (e.g. Rummukainen et al., 2001, Räisänen et al., 2004, Jones et al., 2004, Samuelsson et al., 2011, Kjellström et al., 2011a). The most recent version (RCA4) has been used to produce a large ensemble of simulations for Europe in which the model has been fed with boundary conditions from nine (five) different global climate models under two different forcing scenarios at 50 km (12.5 km) resolution (Strandberg et al., 2014). These RCA4 ensembles allow for climate change studies where in particular robustness related to the choice of boundary conditions (i.e. GCM) can be studied in some detail. The RCA4 ensembles are extensively used at SMHI to produce climate change information used in a Swedish climate service context (e.g. Kjellström et al., 2014) described in this study.

In a European context, EURO-CORDEX (Jacob et al., 2014) recently has produced large ensembles of RCM simulations at approximately 12.5 and 50 km grid spacing to which the RCA4 simulations contribute significantly. These two EURO-CORDEX RCM ensembles are the most comprehensive ones existing to date which makes them particularly suitable for studies of the robustness of future climate change scenario information, and in particular the spread among the ensemble members related to choice of boundary conditions and model formulation in climate change projections at the regional scale. Furthermore, the two different horizontal resolutions allows for further investigation of the added value of high resolution (e.g. Jacob et al., 2014, Kotlarski et al., 2014). These RCM ensembles are now being extensively used for impact studies (e.g. Vautard et al., 2013, Arheimer and Lindström, 2015, Roudier et al., 2015, Pulatov et al., 2016).

In 2005 the Swedish government initiated a Commission on Climate and Vulnerability to “assess regional and local impacts of global climate change on the Swedish society including costs” (SOU, 2007, p.3). This proved to ignite a rapidly increasing interest in user-oriented climate and climate change information that SMHI through the dialogue within the Commission working groups answered to. In December 2007 the Commission presented an overview of the consequences of climate change in Sweden, together with a list of 59 suggestions on how Sweden could proceed with the work (SOU, 2007).

As a result of the conclusions of the Commission, the climate information produced by the Rossby Centre for the Commission was successively made available online to enable usage by counties and municipalities. Apart from climatological maps describing the basic physical parameters (temperature, precipitation, wind, snow, etc.) there were user-oriented indices of frost days, heatwaves and growing degree days etc. (Persson et al., 2007). This new climate information provided the Swedish municipalities and counties with unique national views of possible future changes, and further, the dialogue work by the Commission, involving sector organisations and national authorities, inspired an increasingly reflective dialogue process at SMHI on the needs of the users of climate information. Hence the output from the Rossby Centre RCM simulations has contributed in a fundamental way to map vulnerability to climate change in Sweden, which in turn provided discussion material for dialogues with the users in society.

In this paper we describe currently available information: i) the ensemble of RCA4 simulations for Europe, ii) results for Sweden from this ensemble and iii) development of user needs of climate information from Counties and Municipalities in Sweden and how these needs have influenced the presentations of climate data and climate knowledge that constitutes the climate service provided by SMHI.

## 2 Methods and material

### 2.1 Building climate services – meeting the needs of the users

Here, we go through a number of key activities related to mapping and meeting the needs of the users that has been fundamental for the creation of the climate service described in this paper. A number of studies and enquires have contributed to this process over the years. In essence, collectively these activities make up the method that SMHI as a government agency has used to build the stakeholder dialogue. The mapping has been done through a range of different activities involving collection of facts from reports and inquiries, questionnaires, dedicated workshops and in-depth interviews with key persons at governmental authorities, business corporations, etc. The presentation follows a time line but is not strictly chronological. Supporting the text is Fig. 3 where some of the key elements of this development are outlined together with an overview of the regional climate modelling activities at the Rossby Centre that has provided a fundamental input to the climate services being developed at SMHI.

An early attempt to map vulnerability to climate changes and needs for climate change adaptation in Sweden was presented by Rummukainen et al. (2005). Based on information collected from Swedish governmental authorities, business corporations, sectoral organisations and research funding agencies they showed that there was a general need for better decision support in the form of knowledge about future climate change. This included detailed information about the consequences of climate change on different sectors. Specifically, scientifically based information about probabilities of future climate change was requested. Better coordination of communication activities and coordinated messages was also asked for.

Meeting the growing awareness that Sweden in all respects might not be fully adapted to present day weather and climate extremes, let alone possible future conditions, the governmental Commission on Climate and Vulnerability was organized into three main working groups (WGs) spanning across key societal sectors: WG1, Technical infrastructure and spatial planning; WG2, Agriculture, forestry and the natural environment; and WG3, Health and water resources. A fourth working group WG4, Floodings, major lakes etc. (which worked under a shorter timeline) produced a separate report. Within each working group there were several subgroups. Members of the working groups and subgroups were drawn to represent all major actors (local, regional and national governance entities, professional and trade organisations, academia, government agencies). SMHI participated in several of the groups’ meetings. This turned out to be an arena for intensive and fruitful exchange of knowledge and perspectives. From the SMHI perspective, we were able to convey key information and concepts regarding climate change, climate scenarios to an audience that were interested and receptive. At the same time SMHI deepened its understanding of stakeholders’ perspective on climate change issues in relation to other issues pertaining to the future of the different sectors and governance levels (local – national). From a governmental perspective one of the main outcomes of the Commission was that the County administrative boards were tasked with the regional responsibility for climate change adaptation.

When the County Administrative boards were appointed to coordinate the work of adaptation to climate change in Sweden, after the Climate and energy Bill (Anon., 2008) was in place, SMHI together with the Swedish Geotechnical Institute and other national authorities, collaborated to form arenas and meetings for addressing the needs in each county. These needs have evolved over time and focus has shifted from the need of education in what the climate models can provide, support in mapping vulnerable areas in the counties, general information about sea level rise, and extreme weather event warnings to more specific, knowledge informed needs of climate information. A designated climate information reference group was formed in 2011 by SMHI, with members from the coordinators of adaptation from the County Administrative Boards. The group opened up for a more detailed dialogue on user needs. Examples of needs were to have not only climate variables presented per county, but per climatic region, to get more advanced sea level rise information, to continue development of indices and extremes, and to get support in explaining the climate model outputs to society in general, and planners and colleagues at the county and the municipalities specifically.

In 2012 SMHI was appointed by the Swedish Government to lead the National Knowledge Centre for Climate Change Adaptation. The Knowledge Centre stepped up the activities related to linking science, policy and practice by bringing together decision makers, research organisations, businesses and organisations with an interest in climate change adaptation. As part of the activities in this area the centre is responsible for operating the Swedish Web Portal for Climate Change Adaptation (www.klimatanpassning.se) bringing together 18 different governmental agencies. Communication between these agencies, including climate scientists at SMHI, and representatives for the target groups of the portal, i.e. municipalities and county administrative boards, have led to further development of the provided climate information.

In 2014 SMHI was commissioned by the Swedish Government to produce a Guide for use of climate scenarios. The aim was to facilitate different actors work with climate adaptation. The national authorities’ network around the Climate Adaptation Web Portal, the Climate Adaptation Coordinators at the County Administration Boards and the Swedish Association of Local Authorities and Regions were identified as relevant stakeholders for consultation. Results from a questionnaire and a number of interviews clearly showed the need for a web based guide, but there were also requests for a report in Swedish (Persson et al., 2015) and a leaflet providing a brief and accessible introduction. In summary it was concluded that the guide should be easy to read and brief, but at the same time include many aspects related to climate change and climate adaptation. It was also pointed out that the guide should be easy available through the Swedish Web Portal for Climate Change Adaptation.

An example of further development of climate information relates to requests on having the EURO-CORDEX RCP-scenarios downscaled to finer resolution. The main reason was the need for hydrological studies, which demands a downscaling technique correcting systematic deviations from observed data. In 2014 EURO-CORDEX 0.44°-data was downscaled by the Distribution Based Scaling (DBS) method (Yang et al., 2010) and in 2015 SMHI was commissioned to present analyses for the 21 Swedish Counties, based on the downscaled data. Results are available (in Swedish only) at the SMHI web under the headline “Länsanalyser” (i.e. “County analyses”).

### 2.2 Climate model simulations

The regional climate model RCA4 builds on its predecessor RCA3 (Samuelsson et al., 2011) but has undergone substantial physical and technical changes. In the development of RCA4 the aims included that it should be easily transferable and applicable for any domain worldwide without retuning. To facilitate production of ensembles a further prerequisite was that it should be efficient and user friendly to operate. The technical modifications, changes in physical parameterizations in going from RCA3 to RCA4 and the model performance in hindcast experiments are documented in Strandberg et al. (2014). In parallel to model development also the overall data management including pre- and post-processing has been developed within the European projects IS-ENES1/2 (Déandreis et al., 2014) and CLIP-C (www.clipc.eu).

Here, we focus on the RCA4 simulations performed for the EURO-CORDEX domain at 0.44°, corresponding to c. 50 km grid spacing. Our choice of 50 km resolution over the high-resolution EURO-CORDEX simulations at 12.5 km (0.11°) was made to maximize the number of GCMs (see discussion in Chapter 4.1) and as this is the ensemble that most extensively has been put forward to the users. RCA4 has been given boundary conditions from a total of nine different GCMs (Table 2). From here on this particular ensemble of GCMs is referred to as GCM9. The simulations have been performed for i) 1961–2005 with historical forcing and ii) for 2006–2100 under different Representative Concentration Pathways (RCP) scenarios (Moss et al., 2010). In RCA4, the RCP scenarios are expressed as changes in equivalent carbon dioxide concentrations as interpolated from one year to the next. Here we use three different RCP scenarios that have been assessed in IPCC (2013):

• RCP 2.6: Strategies for reducing greenhouse gas emissions cause radiative forcing to stabilise at 2.6 W/m^2^ before the year 2100. For this particular scenario only three simulations were available (cf. Table 2).
• RCP 4.5: Strategies for reducing greenhouse gas emissions cause radiative forcing to stabilise at 4.5 W/m^2^ before the year 2100.
• RCP 8.5: Increased greenhouse gas emissions mean that radiative forcing will reach 8.5 W/m^2^ by the year 2100.

In addition to the EURO-CORDEX simulations with RCA4, SMHI also presents results from older simulations with RCA3 from the Rossby Centre at the web site. The rationale for this is that users of climate information have shown an interest to compare new climate scenarios to older ones that they have previously worked with. In particular the shift from the older SRES emission scenarios to the newer RCP scenarios was instrumental in this context. These older scenarios, which were produced in the European ENSEMBLES project (van der Linden and Mitchell, 2009) and in the Nordic Climate and Energy Systems (CES) project (Kjellström et al., 2011b), are described in detail in Kjellström et al. (2011a). Fig. 3 gives an overview of regional climate model versions, scenario generations and in which research project context these have been produced over the last two decades at the Rossby Centre.

### 2.3 Climate indices

The indices currently presented at the web services (Table 1) are calculated from daily data that are based either on timestepwise information from the model (i.e. 30 min at 50 km resolution) or on 3-hourly instantaneous information. For each year or season a number is calculated for each model simulation after which ensemble mean and spread (taken as one standard deviation) is calculated.

### 2.4 Observations

In the climate service provided at the web page Swedish observations from SMHI are used. The rationale is to display the interannual variability and possible trends in the observations to which the model results can be compared. A common reflection from the Swedish users of climate change information is that they are interested in relating future climate change to the observed climate including its variability. The observations have been gridded to a 4 × 4 km grid as described by Johansson (2000) and Johansson and Chen, 2003, Johansson and Chen, 2005. This information is only available for areas in Sweden.

## 3 Results

### 3.1 How can the RCA4 ensemble be used to inform about future climate change in Sweden?

A prominent result of the long-term dialogue between users of climate information and SMHI as outlined above is the information provided at the SMHI web page. Here, we present how results from the RCA4 ensemble are displayed there and give a few examples of what the results show in terms of climate change for Sweden. A more extensive material covering all of Europe and more indices can be found in Strandberg et al. (2014) and on the web page.

The choice of maps displayed at the web page has been decided upon in dialogue with the end users as described above. In this dialogue requests for a large number of indices have been put forward. For instance, specific indices developed for farmers and agriculture, on heatwaves, intense rains, storms have all been discussed. Requested indices are shown if it is thought that the climate model can represent the variable in question in a realistic way. This generally requires that climate model output from reanalysis-driven simulations has been compared to observations and that the comparison does not show too large biases.

As an example Fig. 4 shows information about the vegetation period (as defined in Table 1) as it is one of the indices deemed interesting by the users. The Figure first shows the situation in the reference period (1971–2000) as: i) the users are not always familiar with what the reference situation actually looks like and have therefore asked for this information; ii) there is a need to inform about what the model climate looks like as the GCM-driven RCA4 results to some extent may deviate from what is observed. Apart from the reference period the climate change signal is displayed for three different time periods, 2011–2040, 2041-2070 and 2071–2100 representing climate changes in the nearest decades, in the middle of the century and at the end of the century all reflecting different time horizons with different interest to different users depending on their respective planning horizon. The gradual changes with time can easily be seen in these maps and anyone interested can easily compare a certain 30-year period in two different emission scenarios to see what “business-as-usual”, “some reduction in emissions”, “stronger reduction in emissions” would imply for the regional climate change signal.

**Fig. 1:**
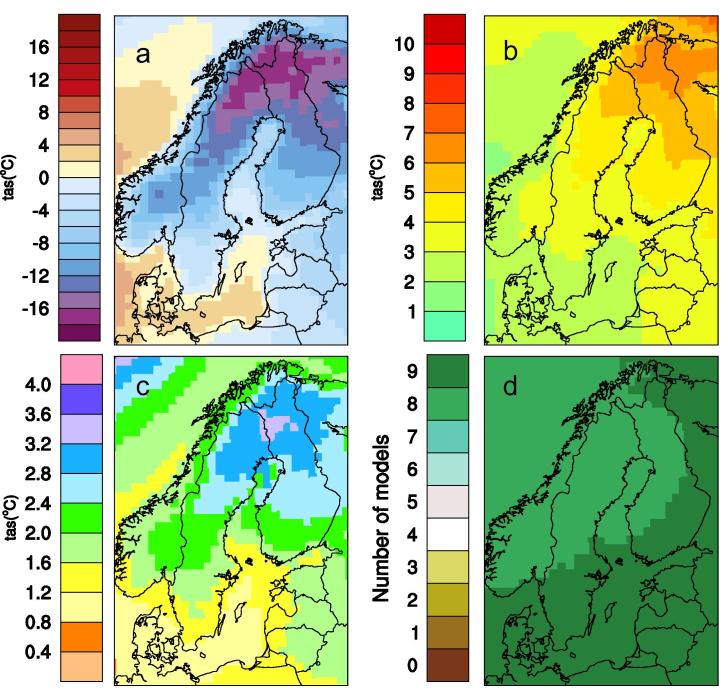
Simulated winter (December-January-February) temperature at the 2 m-level. Each panel shows an ensemble mean of nine RCA4 simulations. Panel (a) shows the average in the reference period (1971–2000) in °C while the other three show aspects of climate change at the latter part of the century (2071–2000) under the RCP4.5 scenario. Panel (b) shows the simulated increase in °C, (c) shows the standard deviation between the nine members (°C), and (d) indicates how many of the nine members that show an increase in temperature.

**Fig. 3:**
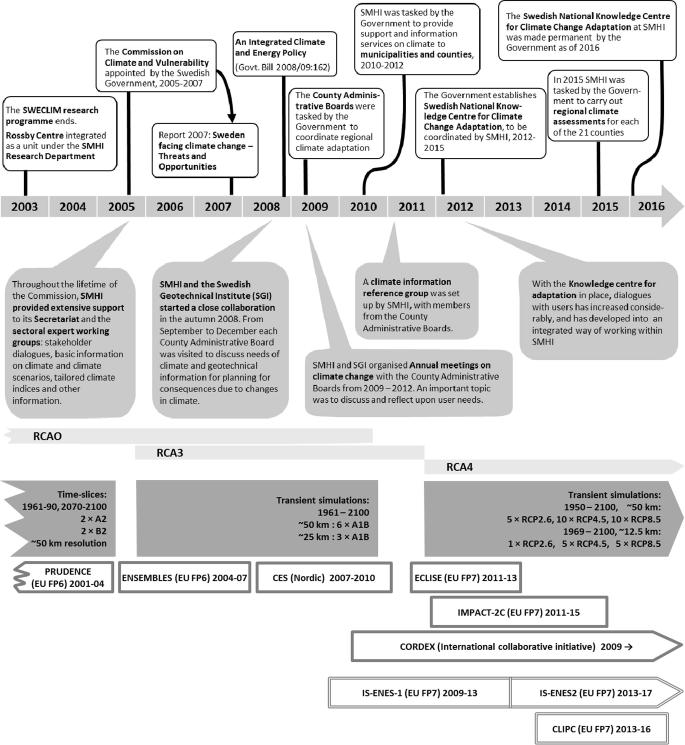
Timeline showing an example of how the user needs dialogue evolved between SMHI and the County Administrative boards, with focus on climate information for adaptation. White boxes (top) denote the tasks which were appointed by the Swedish Government, and the grey boxes explain the activities of SMHI. For simplicity of the diagram only some of the major identified time events are presented. The lower part of the figure illustrates RCA versions (light grey bars) and evolution of RCA-based scenarios at the Rossby Centre (grey fields). In the lowermost part of the figure are mentioned a number of the most important large-scale international projects in which many of the climate scenarios have been produced or where instrumental work with meta-data handling and data publication has been done.

Discussions were also held, both internally at SMHI, and with the coordinators of adaptation at the Counties, on how to best show the robustness and spread (both often referred to as various aspects of the “uncertainty” in climate scenarios, see discussion in Ch. 4.1 below) in a climate model ensemble. During the time from 2005 to 2015 SMHI has moved from presenting time series diagrams showing only a single line representing the ensemble mean to more comprehensive diagrams of output from multi-model ensembles. This includes maps showing multimodel mean of an index and spread between ensemble members indicating robustness in the climate change signal. Fig. 1 illustrates this by showing more information about the climate change signal at a certain period in time (here exemplified for 2071–2100 under the RCP4.5 scenario). Apart from the ensemble mean for the reference period and the ensemble mean change for the scenario period it also shows two maps illustrating spread and robustness of the climate change signal. The spread is calculated as one standard deviation from the ensemble mean based on the nine ensemble members. The robustness criterion is simply calculated by adding the number of models indicating an increase in the index in question. This means that the robustness measure can take any number between 0 and 9 where 9 indicates that all members show an increase and 0 indicates that none of the members show an increase. Both 9 and 0 are therefore connected to robust patterns of climate change concerning sign of the climate change signal. Numbers like 4 or 5 on the other hand, indicates that only half of the ensemble members show an increase, and are consequently associated with less robustness.

In addition to pure model results the dialogue with end users has resulted in the fact that also observations are shown in diagrams at the web page. Fig. 5 illustrates this for temperature in the Stockholm area. For each year the observed anomaly is displayed with a red (positive) or blue (negative) bar representing warmer or colder than average conditions. In the same diagram it can also be studied how the RCA4 ensemble projects changes with time. For this particular application the ensemble mean is shown as well as the maximum and minimum for each year from any of the nine ensemble members. These maximum and minimum numbers illustrates that the model simulates a climate with sometimes warm and sometimes cold years with an interannual variability comparable to the observed one. For this particular case it can be seen that both observations and model results show annual anomalies of up to ±2 °C for the period 1961–1990 (a few years in the model show even larger negative anomalies). For the period 1990–2010 anomalies are mostly on the warm side both in observations and model, and for the future the model indicates that the interannual variability remains similar in amplitude. At the same time the long-term warming trend indicates that even the coldest years at the end of the 21st century are warmer than the warmest years in the reference period.

### 3.2 What does the RCA4 ensemble tell us about future climate change in Sweden?

**Fig. 4:**
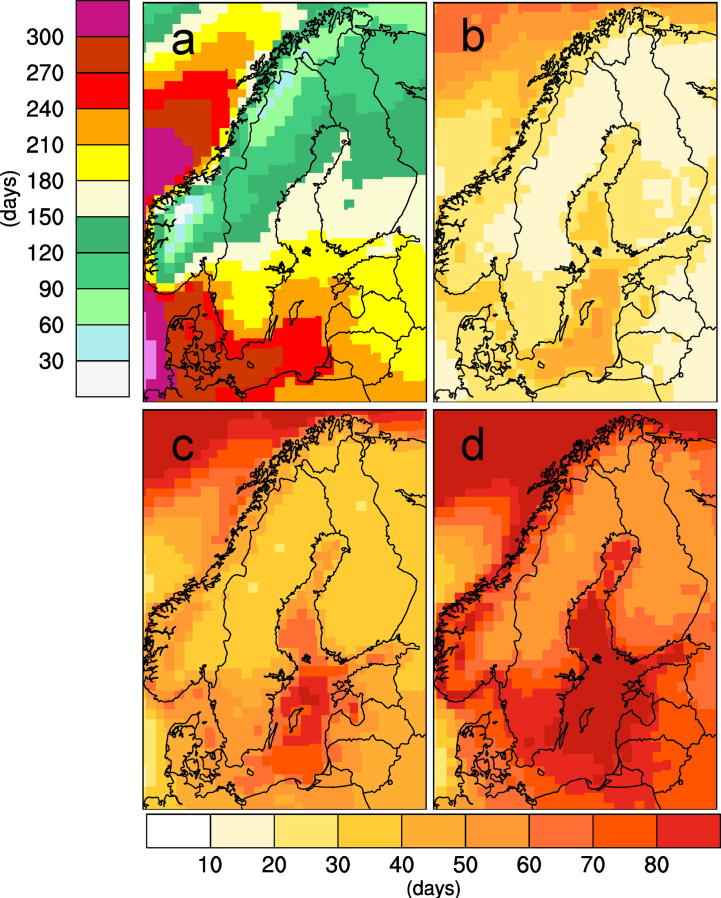
Simulated length of the vegetation period (as defined in Table 1). Each panel shows an ensemble mean of nine RCA4 simulations. Panel (a) shows the number of days as an average in the reference period (1971–2000) while the other three show changes w.r.t. to the reference period for 2011–2040 (b), 2041–2070 (c) and 2071–2100 (d) respectively in the RCP8.5 scenario.

**Fig. 5:**
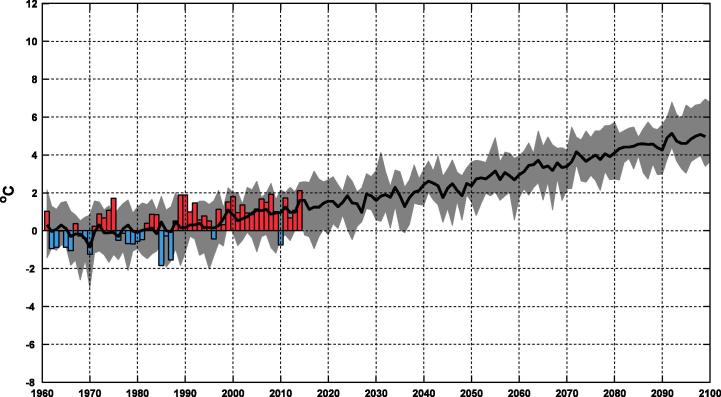
Anomalies in annual mean temperature taken as an area average over the Stockholm County. The blue and red bars represent SMHI observations while the black line (ensemble mean) and grey field (individual maximum and minimum from any ensemble member) are taken from the RCA4 ensemble under the RCP8.5 scenario. (For interpretation of the references to colour in this figure legend, the reader is referred to the web version of this article.)

**Fig. 6:**
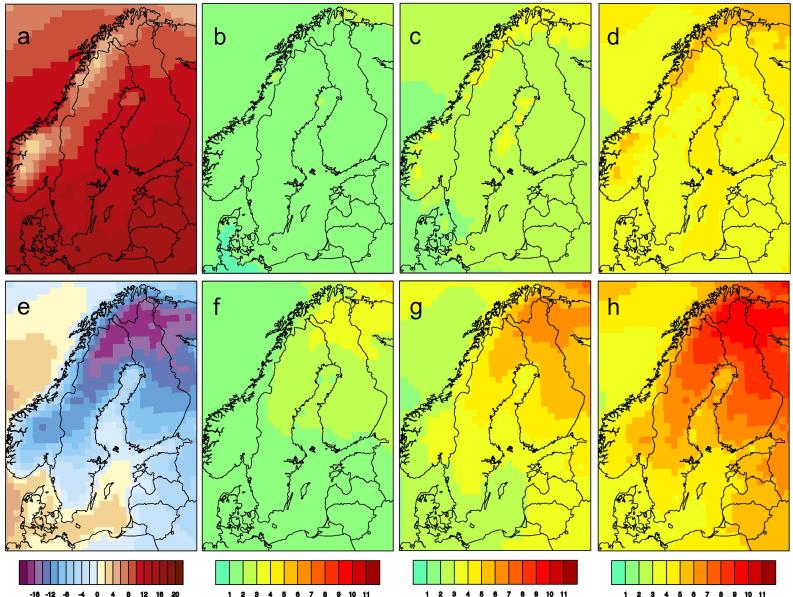
Simulated temperature in summer (a) and winter (e) in the RCA4 nine-member RCP8.5 ensemble for the control period (1971–2000). The other panels show changes w.r.t. the control period for each of the time periods 2011–2040 (b,f), 2041–2070 (c,g) and 2071–2100 (d,h) for summer (b,c,d) and winter (f,g,h). Unit: °C.

The simulated climate in northern Europe undergoes significant changes with increasing global warming. Temperatures increase with time (e.g. Fig. 5) and in all seasons. Fig. 6 shows that the temperature increase is stronger in winter than in summer. This is a consequence of the feedback processes in the climate system related to the reduction in future snow amount and ice extent in the warmer climate (e.g. Kjellström et al., 2011a). Consequently, changes are most pronounced in northern and northeastern parts of the region where extensive snow cover and/or sea ice are prominent features of today’s winter climate. For the same reason the high alpine regions in Norway and along the Swedish-Norwegian border are the regions with largest temperature increases in summer. Taken together, the temperature changes imply that summers are becoming longer and warmer while winters become shorter and milder. Spring and autumn shift in time with the spring season occurring earlier and autumn later. This lengthening of the summer season is clearly illustrated by the change in the vegetation period (Fig. 4). For the reference period the climate model results show strong gradients in the length of the vegetation period from the south to the north and from coastal regions to inland regions. For instance, it can be seen that the simulated vegetation period ranges from being longer than 8 months in the recent past climate in coastal areas in the southernmost parts of Sweden to less than two months in the most extreme parts of the mountainous regions in the north. In line with a gradually warmer climate the vegetation period gets longer and Fig. 4 shows that the geographic patterns of change are very similar over time albeit with a stronger and stronger amplitude. Changes in the temperature climate are highly robust and at the end of the century the different ensemble members all show longer vegetation periods in all of Sweden albeit with somewhat different changes in the number of days (not shown).

**Fig. 7:**
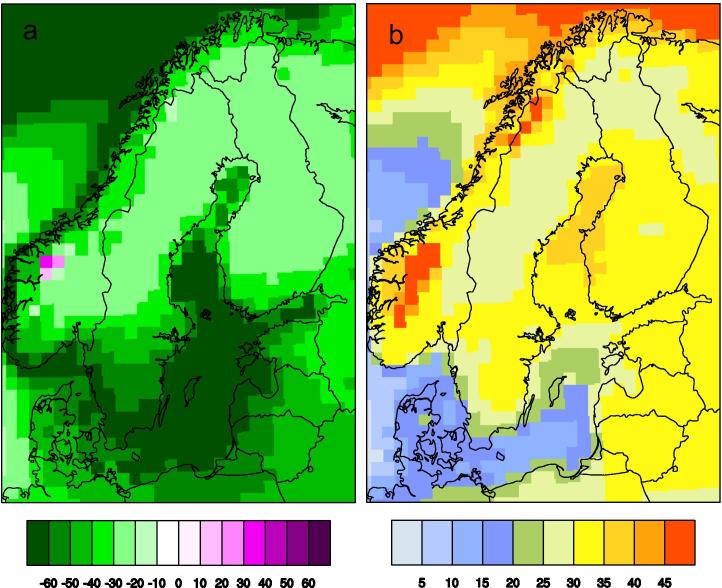
Simulated change in onset (a) and end (b) of the vegetation period (as defined in Table 1). Changes are calculated as the ensemble average of nine RCA4 simulations under the RCP8.5 scenario for 2071–2100 compared to the reference period 1971–2000. Unit: days.

In Sweden the largest changes are seen in southern parts of the country, along the coasts and in the mountain range close to the Norwegian border. Even larger increases are found over the Baltic Sea and parts of the North Atlantic north and west of Northern Scandinavia. A closer look at the vegetation period reveals that it is the changes in spring that are most different in different parts of the country and therefore mainly responsible for the differences in the length of the vegetation period. In fall, the vegetation periods get longer everywhere but with less difference (Fig. 7). Fig. 7 also reveals a few grid points in the highest mountains in Norway where the start of the vegetation period does not become earlier. This is likely a result of the large amounts of snow in these areas that has a profound influence on the temperature climate in spring. In fall, on the contrary, these and other high-altitude regions see the most pronounced prolongation of the vegetation period.

**Fig. 8:**
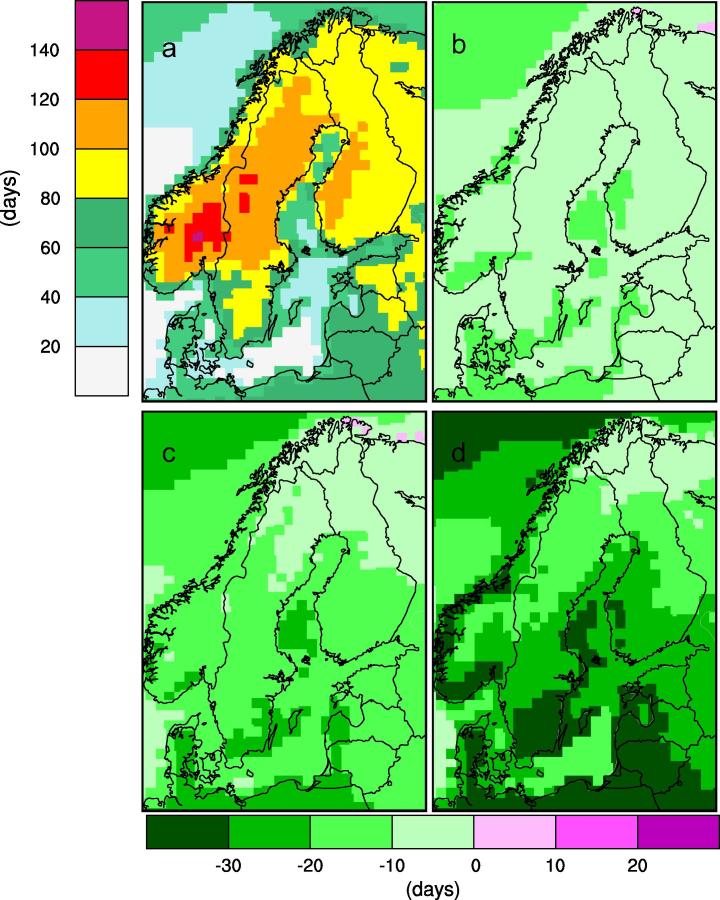
Simulated number of days with zerocrossings (i.e. with temperatures both warmer and colder than 0 °C as defined in Table 1). Each panel shows an ensemble mean of nine RCA4 simulations. Panel (a) shows the number of days as an average in the reference period (1971–2000) while the other three show changes w.r.t. to the reference period for 2011–2040 (b), 2041–2070 (c) and 2071–2100 (d) respectively in the RCP8.5 scenario. Unit: days.

**Fig. 9:**
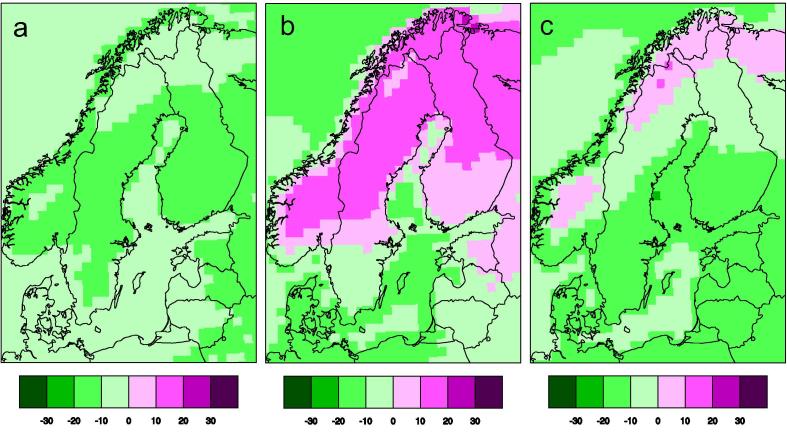
Simulated number of days with zerocrossings (i.e. with temperatures both warmer and colder than 0 °C as defined in Table 1) in autumn (a), winter (b) and spring (c). Changes are calculated as the ensemble average of nine RCA4 simulations under the RCP8.5 scenario for 2071–2100 compared to the reference period 1971–2000. Unit: days.

In parallel with the future warming, the number of days with temperatures remaining within certain intervals changes. Temperatures close to zero degrees, and in particular days when the temperature crosses the zero line, are of particular concern in this context due to their impact on road maintenance traffic conditions (Makkonen et al., 2014), reindeer herding (Jansson et al., 2015) and building constructions (Glaas et al., 2015). On an annual mean basis the number of days with zero-crossings decrease by up to a month or more in the southernmost parts of Sweden in RCP8.5 (Fig. 8). In northernmost Sweden, on the other hand the changes are small. This is a result of a complex change with reduction in the number of days with zero-crossings in spring and autumn while the number is increasing in the winter months (Fig. 9). Clearly, the milder winter conditions lead to changes in the north from the relatively stable cold conditions in today’s climate to a more fluctuating situation with more freeze-thaw cycles. These features are robust over the entire ensemble as all models show the same direction of change even if the actual number of days with zero-crossings differs between the individual ensemble members.

**Fig. 10:**
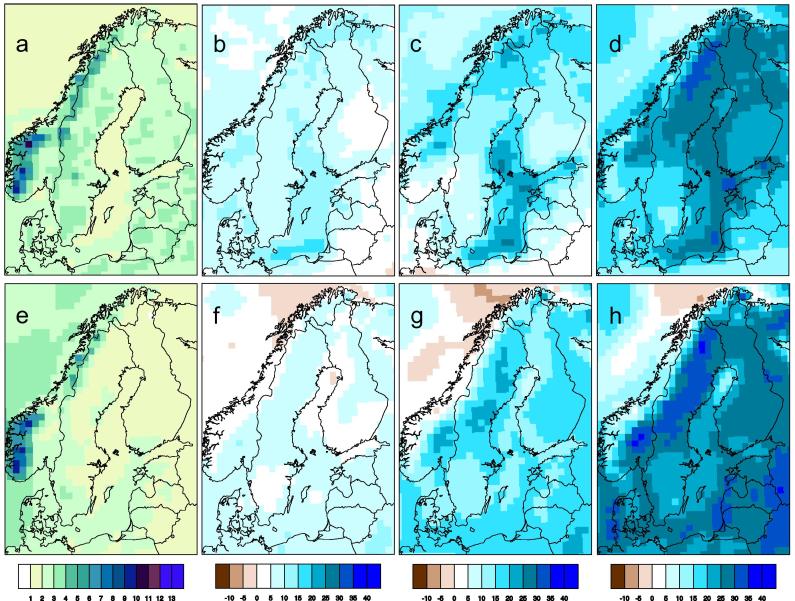
Simulated precipitation (mm/day) in summer (a) and winter (e) in the RCA4 nine-member RCP8.5 ensemble for the control period (1971–2000). The other panels show changes w.r.t. the control period for each of the time periods 2011–2040 (b,f), 2041–2070 (c,g) and 2071–2100 (d,h) for summer (b,c,d) and winter (f,g,h). Unit: %.

**Fig. 11:**
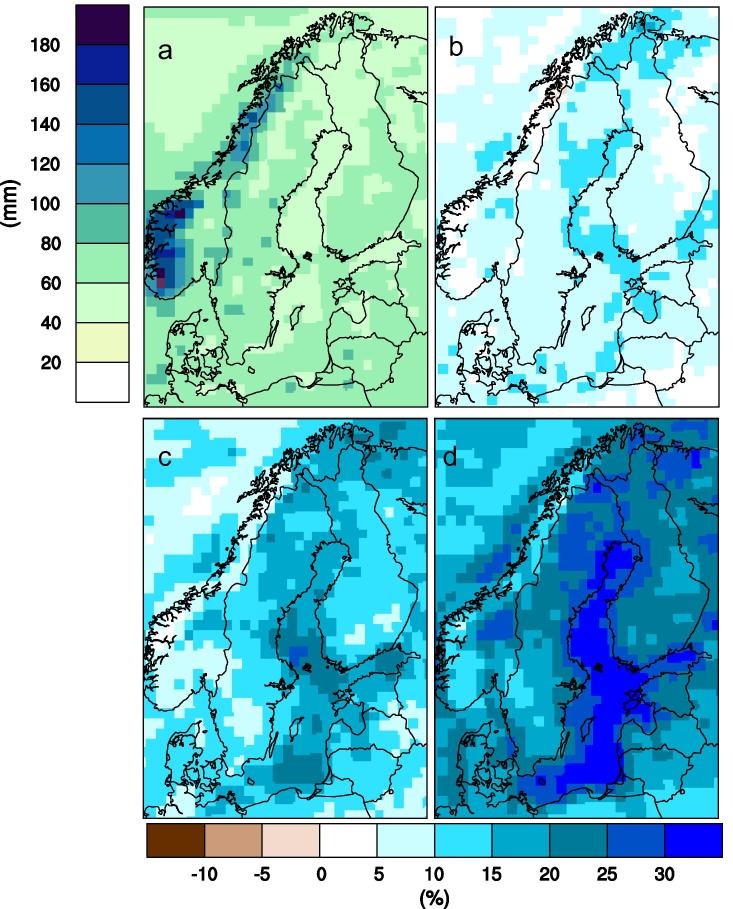
Simulated wet periods (i.e. maximum precipitation accumulated in any consecutive seven-day period as defined in Table 1). Each panel shows an ensemble mean of nine RCA4 simulations. Panel (a) shows the amount (mm) as an average in the reference period (1971–2000) while the other three show changes (%) w.r.t. to the reference period for 2011–2040 (b), 2041–2070 (c) and 2071–2100 (d) respectively in the RCP8.5 scenario.

The strengthened global hydrological cycle resulting from a warmer atmosphere leads to more precipitation in northern Europe (Fig. 10). Increasing changes with time are clear in both winter and summer. In the RCP8.5 scenario ensemble mean changes of more than 25% in large parts of the area are seen in both seasons at the end of the century. Precipitation increases on all time scales, from short-term rain showers up to seasonal, annual and longer periods. Here, we illustrate how wet periods change by looking at how the maximum consecutive seven-day accumulated precipitation increases (Fig. 11). Large amounts of precipitation on such relatively long periods have a strong impact on flooding and thereby on the society. Numerous flooding episodes over the years have caused large damage, both in Sweden and abroad (e.g. Grahn and Nyberg, 2014, Merz et al., 2010). The maps shown in Fig. 11 clearly show the gradual increase in seven-day precipitation in RCP8.5 and at the end of the century the increase can be 20–25% or more in large parts of the country. In the RCP4.5 scenario (not shown) increases are up to 20% and in both scenarios the signal is robust as it can be seen in all ensemble members. It is only in the weaker RCP2.6 scenario that there is some disagreement between the ensemble members in terms of whether they project increases or decreases, and the average change is small in that scenario (not shown here).

## 4 Discussion

### 4.1 Climate change uncertainty

In the scientific discussion about climate scenarios the so called “uncertainty” plays an important role. Therefore, we first discuss our results in this context. Different sources of uncertainty limits the ability to give precise answers about what will happen with the climate in the future. This is true both in a global, and even more in a regional, or local context. The main sources of uncertainty include; i) uncertainty related to future emissions of greenhouse gases and other forcing agents of the climate system, ii) uncertainty related to the response of the climate system to changing forcing conditions, iii) uncertainty related to natural internal variability of the climate system and iv) uncertainty related to the formulation of the climate models we use to produce scenarios for the future. These different sources of uncertainties and their relative role in different temporal and spatial contexts are discussed by Hawkins and Sutton (2009). They conclude that the relative contribution from internal variability is largest in the near future and in a regional perspective, while forcing conditions and climate system response dominates on longer time scales. In a regional climate modelling context, uncertainty related to the choice of GCM has been shown to be one of the most important sources of uncertainty in a longer time perspective (i.e. the second half of this century) in the European PRUDENCE project (Déqué et al., 2007). They found that this was the case most notably for seasonal mean conditions and in particular for temperature, In a shorter time perspective and for other variables and aspects of climate change this may not be the case.

To address uncertainties as listed above climate scientists often use multi-model ensembles consisting of a large number of individual climate simulations (e.g. the CMIP5 multi-model ensemble) that can be used to construct probabilistic estimates of future climate change. This is a powerful tool as it indicates the strength of the evidences for a certain climate change signal. But, as Tebaldi and Knutti (2007) points out, multi-model ensembles that can be used for such calculations do not cover all ranges of uncertainty as they are “ensembles of opportunity”. This means that they rather reflect the specific uncertainty range spanned by the individual models that are included, how many times they have been used, etc. Regardless of this, these multi-model ensembles, constitutes the most comprehensive material existing to date and they are therefore used widely for climate services purposes.

With this background we note that it is more useful for communication with the wider community outside of climate sciences to discuss “spread” and “robustness” of climate scenarios rather than speaking generally about “uncertainty”.

### 4.2 Is the RCA4 ensemble representing spread in an adequate way?

Here, we have used a multi-model ensemble of nine GCMs to provide boundary conditions to the RCM. Even if the ensemble of simulations with RCA4 driven by GCM9 under two different forcing scenarios samples a substantial fraction of all available GCM scenarios in CMIP5 there is still a large number of GCM simulations that are not downscaled by RCA4. A central question therefore relates to how different would the results be if a larger or different ensemble of GCMs where sampled? Another relevant question relates to whether RCA4 changes the climate change signal in any significant way as compared to GCM9?

We first note that the climate sensitivity defined as the transient climate response (TCR) in the GCMs in GCM9 range from 1.3 to 2.5 °C at the time of CO_2_ doubling (Table 2). This range compares well to the estimate put forward in the IPCC assessment of 1.0–2.5 °C (Collins et al., 2013). It can therefore be concluded that GCM9 does represent the spread in TCR and thereby the uncertainty in response of the global mean temperature to a steady increase of the forcing in a 50–100 year time scale as reflected by the CMIP5 GCMs.

**Fig. 12:**
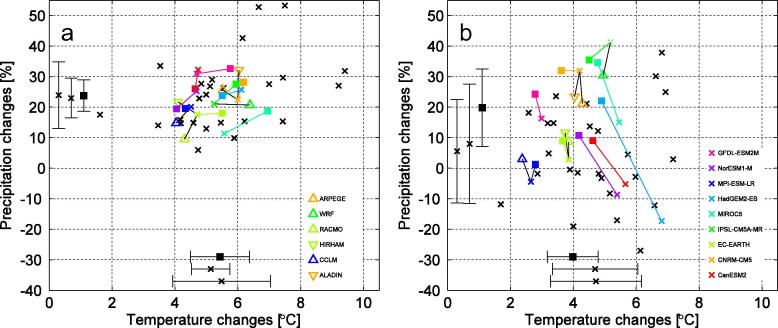
Temperature (°C) and precipitation (%) changes 2071–2100 compared with 1971–2000 in Sweden for winter (a) and summer (b) in RCP8.5. Filled squares represent RCA4 simulations, connected by a line to the corresponding GCM (colour code in the legend of (b), see Table 2 for model names). Black crosses represent other CMIP5 GCMs. Triangles indicate simulations with other RCMs than RCA4 (colour code in the legend of (a), see Table 3 for model names). Mean values and ±1 standard deviations are shown along the x-axis for temperature and the y-axis for precipitation for: the full GCM CMIP5-ensemble (represented by crosses for mean values and dash dotted line for ±1 standard deviation), GCM9 (crosses and full lines) and the RCA4 ensemble (filled squares and dashed lines).

Next, we investigate to what degree GCM9 is representative of a larger GCM-ensemble also in a regional context by comparing GCM9 with 25 other CMIP5 models. Here, we focus on all of Sweden as the coarse resolution in the GCMs precludes any detailed comparisons at regional to local level in Sweden. Fig. 12 shows temperature and precipitation changes as simulated by the 34 CMIP5 GCMs in addition to the nine RCA4 simulations in focus. The spread, defined by the standard deviation, between GCM9 is very similar to that of the larger CMIP5 ensemble, both for temperature and precipitation for summertime conditions. In winter, on the other hand, the GCM9 spread is smaller and, in particular, GCM9 does not contain any outlier, neither in temperature nor in precipitation. Further investigations of the representativity of GCM9 w.r.t. to the larger CMIP5 ensemble can be found in Strandberg et al. (2014). They conclude that GCM9 can be considered to be representative of the full CMIP5-ensemble in several European regions but that another ensemble, using other or more GCMs, could give different, yet similar, results in some regions.

Many of the 34 GCMs shown in Fig. 12 have been run several times sampling the internal natural variability of the climate system as simulated by the models. This implies that the full CMIP5 ensemble is much larger than what has been used here and that there is a strong possibility that some of the members of the total more than 200 individual simulations in CMIP5 show results deviating significantly to what is shown in Fig. 12. Such findings have previously been brought forward by Deser et al. (2012) analysing a 40-member ensemble with one GCM. They concluded that the spread due to internal variability is large and that it differs between different variables with larger spread between simulations associated with precipitation than with temperature. A similar conclusion was drawn by Kjellström et al. (2013) based on RCM results from the ENSEMBLES project. They showed that it takes longer time for changes in precipitation to become statistically significant than it does for changes in temperature implying that the relatively larger natural variability in precipitation masks the climate change signal more efficiently than what is the case for temperature. It is clear that there could potentially be other individual ensemble members showing very different results compared to those presented in Fig. 12. This could involve ensemble members showing weaker but also stronger regional climate change signals over the specific time period in question.

### 4.3 Is RCA4 influencing the spread in any significant way?

The next step is to investigate the influence of RCA4 on the spread. For winter conditions in Sweden the difference between the individual members of GCM9 and RCA4 is relatively small (Fig. 12). It is clear from the figure that the spread is almost the same in RCA4 compared to that in GCM9 for precipitation while it is larger for temperature. In summer RCA4 gives larger increases in precipitation than the GCMs in all but two simulations (the ones where RCA4 is downscaling the CNRM-CM5 and IPSL-CM5A-MR GCMs), and smaller increases in temperature in all but one simulation (MPI-ESM-LR). The spread between the simulations is smaller in RCA4 compared to in GCM9 for both temperature and precipitation. As shown by Strandberg et al. (2014) similar reductions in spread are seen also for other European regions and they hypothesize that this reduction in spread is a consequence of the fact that RCA4 holds one description of the physics of the climate system while each GCM has its own. In this context we note that RCA4 gives either higher temperatures and more precipitation, or lower temperature and less precipitation for winter. Also in summer RCA4 tends to change the signal in a systematic way. Now with less increases in temperature compared to the corresponding GCM in GCM9 and in most cases with increasingly wetter (or less dry) conditions in the future. Notable in this context is that RCA4 changes the signal from a decrease in precipitation of almost 20% in the HadGEM2-ES GCM to an increase of more than 20%, thus changing not only the number but even the sign of the climate change signal in the area.

We also note that another RCM would potentially change the results in another way compared to RCA4. In Fig. 12 we also show results from six other EURO-CORDEX RCMs (Table 3) and it is clear that there are both differences and similarities in the regional response. For instance, in summer most of the RCMs show climate change signals that are within 0.5 °C and 10% from each other although not always changing the GCM signal in the same direction. As none of these other RCMs has downscaled any larger fraction of the CMIP5 GCMs at 0.44° resolution it is difficult to judge whether RCA4 changes results in any systematic way compared to other RCMs. This lack of any larger number of climate projections with other RCMs than RCA4 is the main reason for why the climate service developed at SMHI is based solely on RCA4 and not on a true multi-model ensemble consisting of a large number of GCM-RCM combinations.

## 5 Summary and conclusions

We present how regional climate model scenarios produced at SMHI has been used to provide a climate service to the Swedish society. Focus has been on providing information to the County boards that are responsible for the regional work on climate adaptation. From this work we show that:

• During the process it has become clear that the stakeholders have a need for support in different forms. Explanatory material, in various forms, was therefore developed. Oral presentations as well as dialogues have been necessary tools in the production and design process as part of the iterative approach of forming a climate services.
• The iterative approach of forming a climate services involving both climate scientists and users of climate information has been shown to be successful as the disseminated information has been broadly used and serves as an important source of information for further work on adaptation to climate change in Sweden.
• The climate information put forward here builds on a large number of recent regional climate model simulations and we present results for a number of climate indices for which there is a growing interest from users of climate information. We conclude that the results can be used to address questions on climate change and some of its impacts for Swedish conditions.
• The GCM9 ensemble is a good representative of the wider CMIP5 GCM-ensemble in terms of sampling the spread in regional climate change in Sweden. This implies that the RCA4-ensemble can be used to illustrate uncertainties and robustness in climate change in Sweden.
• RCA4 changes results compared to GCM9, sometimes in a systematic way. Larger ensembles with other RCMs are needed to further examine whether these changes are purely model-dependent or a result of the higher resolution in RCA4 compared to the GCMs.

## Figures and Tables

**Fig. 2:**
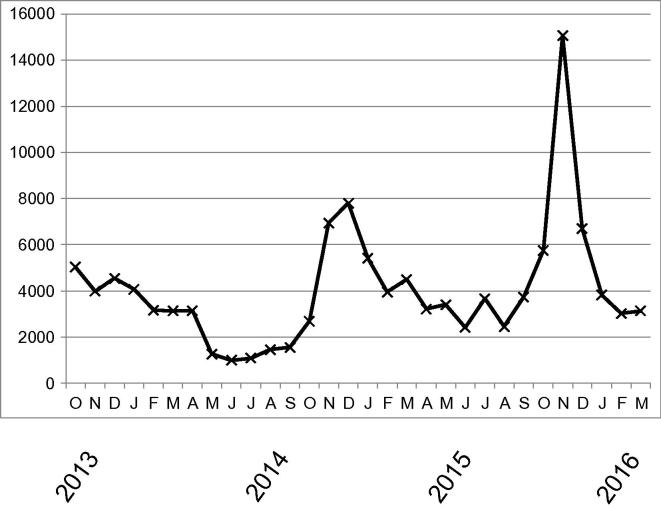
Number of times that the web service (http://www.smhi.se/klimat/framtidens-klimat/klimatscenarier) has been accessed as a function of time between October 2013 and March 2016. Source of information Google Analytics (25 April 2016).

**Table 1:** Climate indices presented at www.smhi.se derived from the RCA4 50 km ensembles.

Parameter	Climate indices	Description
Temperature	Mean, minimum and maximum temperature	Seasonal means based on daily averages calculated from 3-hourly data
Vegetation period	Length, Start day and End day	The vegetation period is defined as days in a year when the daily mean temperature exceed 5 °C. Single warm days in winter have been left out by starting the vegetation period only in the first period with at least 4 consecutive days with temperatures exceeding 5 °C
Zerocrossings	Number of days	Number of days when the temperature is both below and above 0 °C during parts of the day. Information from the model from each time step
Spring frost	Last day in spring with frost	The last day in spring when the temperature is below 0 °C during some part of the day
Precipitation	Monthly sum, maximum daily amount	Daily precipitation is accumulated over all time steps in the 24-hourperiod
Heavy precipitation	Number of days with heavy precipitation	Number of days with more than 10 mm precipitation. Can be considered heavy precipitation in a climate model context, not in single point observations
Wet period	Yearly maximum weekly precipitation	Maximum of consecutive 7-day running sum precipitation
Dry period	Longest dry period in a year	Longest period with less than 1 mm/day in any day
Wind speed	Maximum yearly gust wind speed	Strongest wind in a year, based on 30-min data from the model

**Table 2:** List of CMIP5 GCMs that have been used to provide boundary conditions for the RCA4 runs presented here. Transient climate response (TCR) is defined as the immediate temperature change after a doubling of atmospheric CO_2_ (Cubash et al., 2001). The rightmost column indicates which RCP scenarios that have been run (2 – RCP4.5 and 8.5, 3 – RCP 2.6, 4.5 and 8.5).

No	Modelling centre	Model name	References	TCR (°C)	RCP
1	Canadian Centre for Climate Modelling and Analysis	CanESM2	Chylek et al. (2011)	2.4	2
2	Centre National de Recherches Météorologiques/Centre Européen de Recherche et Formation Avancée en Calcul Scientifique	CNRM-CM5	Voldoire et al. (2012)	2.1	2
3	EC-EARTH consortium	EC-EARTH	Hazeleger et al. (2010)	2.0	3
4	NOAA Geophysical Fluid Dynamics Laboratory	GFDL-ESM2M	Dunne et al. (2012)	1.3	2
5	Met Office Hadley Centre	HadGEM2-ES	Collins et al. (2011)	2.5	3
6	Institut Pierre-Simon Laplace	IPSL-CM5A-MR	Dufresne et al. (2013)	2.0	2
7	Atmosphere and Ocean Research Institute (The University of Tokyo), National Institute for Environmental Studies and Japan Agency for Marine-Earth Science and Technology	MIROC5	Watanabe et al. (2011)	1.5	2
8	Max Planck Institute for Meteorology	MPI-ESM-LR	Popke et al. (2013)	2.0	3
9	Norwegian Climate Centre	NorESM1-M	Bentsen et al. (2013)	1.4	2

**Table 3:** List of EURO-CORDEX simulations with other RCMs than RCA4 that have been used to illustrate the climate change signal over Sweden in Fig. 12. GCMs are as listed in Table 2.

No	Modelling centre	RCM name	GCM	RCM reference
1	Centre National de Recherches Météorologiques	ALADIN	CNRM-CM5	Colin et al. (2010)
2	Centre National de Recherches Météorologiques	ARPEGE	CNRM-CM5	Déqué et al. (1994)
3	Danish Meteorological Institute	HIRHAM	EC-EARTH	Christensen et al. (1998)
4	Royal Netherlands Meteorological Institute	RACMO	EC-EARTH	Meijgaard et al. (2012)
5	National Center for Atmospheric Research	WRF	IPSL-CM5A-MR	Skamarock et al. (2008)
6	Consortium for Small-scale Modeling	CCLM	MPI-ESM-LR	Rockel et al. (2008)
